# Associations between Infant Feeding Practices and Length, Weight, and Disease in Developing Countries

**DOI:** 10.3389/fped.2013.00021

**Published:** 2013-09-04

**Authors:** Benjamin O. Yarnoff, Benjamin T. Allaire, Patrick Detzel

**Affiliations:** ^1^Public Health Economics Program, RTI International, Research Triangle Park, NC, USA; ^2^Nestlé Research Center, Lausanne, Switzerland

**Keywords:** infant health, infant nutrition, feeding practices, infant development, developing countries

## Abstract

The health benefits of exclusive breastfeeding are well-known, but the relative detrimental impacts of other foods on infant health are unknown. Because infants in developing countries are fed a wide range of food, quantifying the burden of these diverse feeding practices on infant health is essential for public health policy. We used data from the Demographic Health Survey from 20 developing countries over multiple years to examine the independent association of six different types of food (exclusive breastfeeding, non-exclusive breastfeeding, infant formula, milk liquids, non-milk liquids, and solid foods) with five measures of infant health (length, weight, diarrhea, fever, and cough). We estimated associations with regression analysis, controlling for confounding factors with infant, mother, and household factors and community-year fixed effects. We used these estimates in a simulation model to quantify the burden of different combinations of food on infant health. We show that for an infant younger than 6 months old, following current guidelines and exclusively breastfeeding instead of giving the infant solid foods may increase length by 0.75 cm and weight by 0.25 kg and decrease diarrhea, fever, and cough prevalence by 8, 12, and 11%, respectively. We found that the burden on infant health of some feeding practices is less than others. Although all other feeding practices are associated with worse health outcomes than exclusive breastfeeding, breastfeeding supplemented with liquids has a lower burden on infant health than solid foods and infant formula has a lower burden than milk or non-milk liquids as measured by four of five health metrics. Providing specific quantified burden estimates of these practices can help inform public health policy related to infant feeding practices.

## Introduction

The infant health benefits of exclusive breastfeeding are well-known. Studies have linked exclusive breastfeeding to a reduced risk of diarrhea (1), lower respiratory infections (2), asthma (3), stunting (4, 5), and mortality (2, 6) as well as improved motor development (7) for infants in developing countries. Breastfeeding promotion programs have been shown to reduce infant mortality and stunting (8). Several meta-analyses and evidence reviews have confirmed these findings: exclusive breastfeeding remains the best option for a child’s first 6 months (9). As a result, the World Health Organization (WHO) and UNICEF recommend exclusive breastfeeding to 6 months of age, with continued breastfeeding along with appropriate complementary foods to 2 years of age or beyond (10).

However, when examining infant health in developing countries, focusing solely on breastfeeding or exclusive breastfeeding ignores the complexity of infant feeding that may be important for quantifying the burden of non-exclusive breastfeeding on infant health. It has been shown that infants in developing countries receive a wide range of foods even when breastfed. For example, 22% of mothers in surveys from 20 developing countries feed their infants solid foods before 6 months of age (11). Different types of foods may have different effects on infant health, so it is important to examine a wider range of feeding when examining the burden on infant health. Past research on the effects of feeding on infant health has largely ignored this complexity and focused solely on breastfeeding (1–5), with a few exceptions for complementary feeding in older infants. One study of infants older than 6 months constructed a composite scoring system for the nutritional value of feeding that ranges from 0 to 12 (e.g., breastfeeding at 6–9 months increased the score by 2, and using a variety of complementary foods increased the score by 1–2) and found poor feeding practices (low scores) were associated with stunting across seven Latin American countries (12). Food diversity among older infants has also been shown to improve anthropometric measures in other developing countries (13). However, a study of children aged 6–24 months in Senegal using the same methods, found no association between diet and anthropometric measures (14). Another study examined infant feeding practices in Kenya and reported that infants were fed a variety of non-breast milk liquids and solids at 6 and 10 weeks. However, anthropometric measures in these infants was not statistically different from the WHO reference group (15). A small study in Mexico examined difference between breastfed and formula fed infants and found an association between breastfeeding and reduced acute respiratory infection (16).

This study is the first to systematically quantify the association between a range of infant feeding practices and infant health for a large and geographically diverse sample of infants. We examine the association of six types of feeding (exclusive breastfeeding, non-exclusive breastfeeding, infant formula, milk liquids, non-milk liquids, and solid foods) with five health outcomes (length-for-age *z*-score, weight-for-length *z*-score, diarrhea, fever, and cough) for infants in two age groups (less than 6 months and 6 months to 1 year) across 20 developing countries. We use these estimates in a simulation model to quantify the burden of different combinations of food on infant health. Furthermore, unlike previous work, we control for most potentially confounding factors with community-year fixed effects (a type of hierarchical model) and infant, mother, and household controls. By controlling for these confounders, we can produce better estimates of the association between feeding choices on infant health in developing countries.

## Materials and Methods

We compared the health of infants receiving six types of food, using econometric techniques to identify the association of each food type with infant health. We used these estimates in a simulation model to estimate the burden of different combinations of food on infant health.

### Data sources

We used publicly available data from the Demographic Health Survey (DHS) for multiple survey years from 20 developing countries (see Table A1 in Appendix for the full list of countries and years). We decided *ex ante* to examine 20 countries from the Africa, Asia, Latin America regions and selected these countries based on the following criteria: multiple survey years per country and the presence of information on infant feeding and maternal characteristics in each survey. Countries were selected to maximize these two criteria while also maintaining a distribution across the regions. We tested the subsample for the representativeness of the full sample of DHS surveys by comparing mean measures of breastfeeding for infants age 0–6 months, under-5 mortality, and diarrhea incidence between our subsample and the full sample. Mean indicators were virtually identical between this subsample and the full sample of DHS surveys for exclusive breastfeeding among infants 0–6 months old (35% in the full sample and 33% in the subsample), non-exclusive breastfeeding among infants 0–6 months old (61% in the full sample and 63% in the subsample), under-5 mortality in the past five years (99 per 1,000 in the full sample and 101 per 1,000 in the subsample), and diarrhea incidence (20% in the full sample and 21% in the subsample). These similarities suggest that this subsample is representative of overall feeding practices in the full DHS sample and that results are generalizable across the full geography of the DHS.

We pooled multiple years of cross-sectional data from the 20 sample countries. The DHS is a nationally representative survey of women aged 15–49 that is funded by USAID, receives technical support from the US Census Bureau, and is conducted in developing countries approximately every 5 years. The survey asks about the health of each woman and her children as well as demographics and socioeconomics. Importantly, the DHS asks about all foods that the woman’s youngest child was given in the past 24 h. Our sample consists of 72,566 infants divided across two age groups: 0–6 months (37,750 infants) and 6 months to 1 year (34,816 infants).

### Variables

The independent variables of interest are what types of food an infant is fed. We used data from the 24-h infant food recall portion of the DHS to create indicator variables for six types of food: exclusive breastfeeding, non-exclusive breastfeeding, infant formula, milk liquids, non-milk liquids, and solid foods. The food recall variables that went into each feeding type definition are given in Table A2 in Appendix. Each of these indicator variables is equal to one if the infant received that type of food in the past 24 h and zero otherwise. An infant was categorized as being exclusively breastfed if he or she was breastfed in the past 24 h, but did not receive any other types of food. This construction measures only whether the infant was exclusively breastfed in the past 24 h rather than on a regular basis, but is the best measure available. Note that the feeding types other than exclusive breastfeeding are not mutually exclusive so that an infant may receive, for example, both infant formula and milk liquids or both non-exclusive breastfeeding and solid foods. In some cases, a mother was not asked about one or more types of food. In those cases, we used the common missing data imputation technique of including an extra indicator variable that is equal to one if the feeding type is missing and zero otherwise. Other independent variables used as controls are mother’s recall of relative infant size at birth (indicator variables for very small, smaller than average, average, larger than average, and very large), infant vaccination history (indicator variable for whether the infant has received any vaccination), infant gender, maternal education (indicator variables for no formal education, incomplete primary, complete primary, incomplete secondary, complete secondary, and post-secondary), maternal work status (indicator variable for whether she works outside the home), maternal occupation (indicator variables for no occupation and nine occupation categories), maternal weight (in kilograms), maternal height (in centimeters), number of household members, number of children in the household, and whether the family has agricultural land.

The dependent variables of interest are infant length-for-age *z*-score, infant weight-for-length *z*-score, whether the infant had diarrhea in the past 2 weeks, whether the infant had a fever in the past 2 weeks, and whether the infant had a cough in the past 2 weeks. These are the primary measures of health available in the data. Infant length-for-age *z*-score and infant weight-for-length *z*-score are continuous variables that represent the number of standard deviations that the value of length-for-age and weight-for-length falls from the reference mean. These variables can range from -3.99 to 3.99 in value. Infant diarrhea, fever, and cough are each indicator variables equal to one of the infant had the symptom in the past 2 weeks and zero otherwise.

### Statistical methodology

To estimate associations between food types and infant health, it is essential to control for the large number of confounding factors that may bias estimates. Three categories of potential confounding factors are included in this analysis: infant-specific factors, community resources and geographic factors, and household resources and behaviors.

We controlled for many of these factors by including mother’s recall of relative infant size at birth. This variable represents the combined effect the infant’s genetic health endowment and all health inputs given to an infant *in utero* such as maternal nutrition and care (7, 17). This is an important control variable because of the correlation over time between infant health and infant health inputs.

We controlled further for all community resources and geographic factors by including community-year fixed effects (a type of hierarchical modeling). Community is defined by the primary sampling unit from the DHS. Practically, these fixed effects are estimated by including dummy variables for each community and year in the data. This holds constant all factors specific to each community in a given year so that estimates are not confounded by community factors. They are included to control for all community factors not observed in our data, including availability of health resources and types of food, geographic disease and agriculture characteristics, and weather history. This is of special concern here, because we are using such a wide range of countries and years. Because these factors are likely to bias estimates by influencing both infant health and types of food given to the infant, these fixed effects are needed to isolate the impacts of feeding on infant health. This is the first study of the association between feeding and infant health to include community-year fixed effects.

We controlled for household resources and behaviors by including mother’s health (weight and height) and socioeconomic characteristics such as education, work status, occupation, weight, height, number of household members, number of children in the household, and whether the family has agricultural land. Finally, we controlled for household health-seeking behavior by controlling for whether the infant has received any vaccinations. This is not a complete set of household controls, but represents what is available in the data. Sensitivity analysis using the included variables (not reported) provides support that this set of variables is sufficient to control for household resources and behaviors.

We estimated a fixed effect regression (a type of hierarchical model) with the control variables outlined above using DHS sample weights. We estimated the regressions separately for two age groups: less than 6 months old and 6 months to 1 year old. For estimates of disease outcomes, we tested these estimates against Logistic estimates and found that the two estimation techniques produced qualitatively similar results. In addition to the standard *p*-value threshold of 0.05 used to identify statistical significance, we identify marginal statistical significance as *p*-value less than 0.10, because of the reduced statistical power produced by the large number of community-year fixed effects in the analysis.

This regression analysis provides estimates of the association with infant health of exclusive and non-exclusive breastfeeding relative to no breastfeeding and the association with infant health of each food type conditional on not exclusively breastfeeding. However, a feeding practice consists of a combination of these food types. Therefore, to quantify the burden of a feeding practice, we use these regression estimates to simulate the predicted impact of several combinations of food and compare them to the counterfactual of exclusive breastfeeding. We use the regression coefficients to predict health levels for four different behaviors: exclusive breastfeeding; non-exclusive breastfeeding supplemented with solids; non-exclusive breastfeeding supplemented with liquid milk; and non-exclusive breastfeeding supplemented with infant formula. The burdens of feeding practices were estimated as the difference between exclusively breastfeeding, the WHO recommended feeding behavior, and other, non-exclusively breastfeeding practices. We also compared these inappropriate feeding types to each other to evaluate their relative burdens. All estimates were conducted using the sample-weighted averages of the covariates in the regression and were bootstrapped 1,000 times to provide 95% confidence intervals.

Stata (version 11.2) was used to perform all statistical analysis.

## Results

Infants in both age groups are fed a wide range of foods (Table 1). For infants younger than 6 months, only 33% were exclusively breastfed while 63% were non-exclusively breastfed and 4% were not breastfed at all. Of the last group, most received a range of substitutes, predominantly non-milk liquids and infant formula. Only 4% of this small group was exclusively fed infant formula. Use of other feeding types is broadly distributed, with the highest concentration in non-milk liquids. For infants between 6 months and 1 year old, 4% were exclusively breastfed, 87% were non-exclusively breastfed, and 9% received no breast milk at all. Use of the other feeding types is again broadly distributed, with the highest concentration in non-milk liquids and solid foods.

**Table 1:** **Food use by age group**.

Variable	Age < 6 months	Age 6 months to 1 year
**BREASTFEEDING**		
Exclusive breastfeeding	0.33	0.04
Non-exclusive breastfeeding	0.63	0.87
**FEEDING TYPE IF NON-EXCLUSIVELY BREASTFEEDING**		
Infant formula	0.19	0.14
Milk liquids	0.27	0.33
Non-milk liquids	0.88	0.95
Solid foods	0.39	0.82
**FEEDING TYPE IF NO BREASTFEEDING**		
Exclusive infant formula	0.04	0.00
Non-exclusive infant formula	0.60	0.55
Milk liquids	0.54	0.69
Non-milk liquids	0.85	0.96
Solid foods	0.60	0.94

As expected, for infants younger than 6 months, we found that exclusive breastfeeding has a positive association with length and weight and a negative association with diarrhea incidence, although these estimates are only marginally statistically significant (Table 2). Non-exclusive breastfeeding has similar associations but the magnitude is not nearly as pronounced. Solid foods, in contrast, have a negative association with weight and a positive association with diarrhea, fever, and cough incidence. Milk liquids, as well, have a negative association with weight, but no other health measure. Infant formula and non-milk liquids do not have statistically significant associations with infant health.

**Table 2:** **Association of six types of foods with infant health, age < 6 months**.

Feeding type	Length *z*-score	Weight *z*-score	Diarrhea	Fever	Cough
**BREASTFEEDING: REFERENCE GROUP = NO BREASTFEEDING**					
Exclusive breastfeeding	0.31*	0.24*	−0.07*	−0.06	−0.03
	(0.16)	(0.13)	(0.04)	(0.05)	(0.05)
Non-exclusive breastfeeding	0.16	0.23*	−0.05	−0.01	0.01
	(0.14)	(0.12)	(0.04)	(0.05)	(0.04)
**OTHER FOODS: REFERENCE GROUP = NOT RECEIVING A GIVEN FOOD**					
Infant formula	−0.10	−0.05	0.00	0.00	0.01
	(0.07)	(0.07)	(0.02)	(0.02)	(0.03)
Milk liquids	−0.01	−0.10**	−0.02	0.02	0.02
	(0.05)	(0.04)	(0.01)	(0.02)	(0.02)
Non-milk liquids	0.03	−0.04	0.02	0.01	0.00
	(0.06)	(0.06)	(0.02)	(0.02)	(0.02)
Solid foods	0.01	−0.16**	0.06**	0.08**	0.07**
	(0.04)	(0.04)	(0.01)	(0.01)	(0.01)
Observations	37.750	37.214	37.719	36.533	37.372

All regressions include controls for infant size at birth, infant vaccination history, infant gender, maternal education, maternal work status, maternal occupation, maternal weight, maternal height, number of household members, number of infants in the household, whether the family has agricultural land, indicator variables for missing feeding type, and community-year fixed effects. Robust standard errors (clustered on community) are in parentheses. *p < 0.1, **p < 0.05.

For infants age 6 months to 1 year, no feeding type has a statistically significant association with infant health that is consistent across health measures (Table 3). Unlike in infants age 0–6 months, the consumption of solid foods has a mixed association: positive for length, but negative for weight. Non-exclusive breastfeeding has a negative association with length, but no association with any other measure of health.

**Table 3:** **Association of six types of foods with infant health, age 6 months to 1 year**.

Feeding type	Length *z*-score	Weight *z*-score	Diarrhea	Fever	Cough
**BREASTFEEDING: REFERENCE GROUP = NO BREASTFEEDING**					
Exclusive breastfeeding	−0.18	0.01	−0.03	−0.03	−0.07
	(0.16)	(0.13)	(0.04)	(0.05)	(0.05)
Non-exclusive breastfeeding	−0.21**	0.01	−0.02	−0.03	−0.04
	(0.10)	(0.08)	(0.03)	(0.03)	(0.03)
**OTHER FOODS: REFERENCE GROUP = NOT RECEIVING A GIVEN FOOD**					
Infant formula	0.04	−0.10	−0.02	−0.01	0.03
	(0.07)	(0.07)	(0.02)	(0.02)	(0.02)
Milk liquids	0.03	−0.04	−0.01	−0.01	0.00
	(0.04)	(0.04)	(0.01)	(0.01)	(0.01)
Non-milk liquids	0.04	0.02	0.01	−0.00	−0.02
	(0.08)	(0.07)	(0.02)	(0.03)	(0.03)
Solid foods	0.08*	−0.07*	0.01	0.00	−0.01
	(0.05)	(0.04)	(0.01)	(0.02)	(0.02)
Observations	34.816	34.304	34.796	33.723	34.484

All regressions include controls for infant size at birth, infant vaccination history, infant gender, maternal education, maternal work status, maternal occupation, maternal weight, maternal height, number of household members, number of infants in the household, whether the family has agricultural land, indicator variables for missing feeding type, and community-year fixed effects. Robust standard errors (clustered on community) are in parentheses. *p < 0.1, **p < 0.05.

Simulation of predicted health outcomes under different food combinations shows that the burden of non-exclusive breastfeeding is different for different combinations of food types (Table 4). With the exception of five outcomes, there are positive and statistically significant health benefits from exclusive breastfeeding. However, these benefits vary by different types of alternative feeding practices. The feeding practice with the greatest burden is non-exclusive breastfeeding supplemented with solid food. The three liquid food types all have a lower estimated burden. Infant formula has the lowest burden (or tied by statistical insignificance) of the three as measured by weight, diarrhea, incidence, fever, and cough. Auxiliary simulations (not reported here) testing the statistical significance of differences between inappropriate feeding practices indicate that supplementing with solid foods is associated with significantly worse disease incidence than infant formula, non-milk liquids, or milk liquids. Differences between supplementation with infant formula and supplementation with milk liquids or non-milk liquids are not significant however.

**Table 4:** **Simulated impact of changing to exclusive breastfeeding on infant health, age < 6 months**.

Original feeding type	Length *z*-score	Weight *z*-score	Diarrhea	Fever	Cough
NEBF + Solids	0.138**	0.174**	−0.083**	−0.124**	−0.107**
	(0.022, 0.255)	(0.060, 0.288)	(−0.115, −0.050)	(−0.164, −0.084)	(−0.150, −0.064)
NEBF + Liquid milk	0.160**	0.115**	−0.006	−0.068**	−0.059**
	(0.055, 0.266)	(0.012, 0.218)	(−0.033, 0.022)	(−0.100, −0.036)	(−0.097, −0.022)
NEBF + Non-milk Liquid	0.119**	0.058	−0.046**	−0.062**	−0.040**
	(0.056, 0.182)	(−0.018, 0.135)	(−0.062, −0.030)	(−0.080, −0.043)	(−0.060, −0.020)
NEBF + Formula	0.249**	0.064	−0.028	−0.052**	−0.050
	(0.098, 0.401)	(−0.083, 0.212)	(−0.069, 0.013)	(−0.101, −0.003)	(−0.104, 0.004)

NEBF, non-exclusive breastfeeding. Confidence intervals are presented in parentheses and based upon 1,000 bootstrapped replications of regression analysis.**p < 0.05.

## Discussion

Consistent with previous evidence, we show that a large percentage of mothers do not follow current guidelines for feeding infants: only 33% of mothers of infants younger than 6 months breastfeed exclusively (11). We find that these feeding decisions place a substantial burden on infant health and that some feeding practices have a greater burden than others. The burden is concentrated in infants at younger ages, where growth and brain development are crucial to an infant’s long-term development.

Using estimates of the association between food types and infant health, we provide quantified estimates of the relationship between different feeding practices and reported infant health outcomes. For example, for an infant younger than 6 months in our sample, we find that if he or she were to be fed following current guidelines and exclusively breastfeed instead of supplementing with solid foods, the infant may be 0.75 cm longer and 0.25 kg heavier (based on estimated *z*-scores). Furthermore, following current WHO breastfeeding guidelines instead of supplementing with solid foods is associated with a substantial reduction in the likelihood of diarrhea, fever, and cough. This result confirms previous findings: exclusive breastfeeding remains the best option for children up to 6 months of age. We also show that the burden of some feeding practices is less than others. Although all other feeding practices are associated with worse health outcomes than exclusive breastfeeding, breastfeeding supplemented with liquids has a lower burden on infant health than solid foods and infant formula has a lower burden than milk or non-milk liquids by four of the five infant health measures. Identifying this hierarchy is important, because there may be mothers who for some reason are unable or unwilling to exclusively breastfeed.

Unlike for infants younger than 6 months, we find no consistent evidence of a relationship between food types and infant health for infants between the ages of 6 months and 1 year. The few statistically significant associations are either mixed in sign (solid foods) or for only one health measure (non-exclusive breastfeeding). These results accord with intuition as infants at this age are being introduced to a wide variety of foods at varying times and the best feeding practices are less well defined. Exclusive breastfeeding does not have a statistically significant association with infant health for this age group.

Our study has several limitations. First, although we have indicators for multiple feeding behaviors, we lack important data on the intensity of each type of feeding. Second, our data consist of multiple cross-sections, but we lack the ability to track households across time. Longitudinal data would allow for more precise estimation of the feeding decisions on infant health. This analysis attempts to provide unbiased estimates of the association of feeding types with infant health by controlling for infant, mother, and household factors and community-year fixed effects. This approach arguably controls for much of the potential bias from confounding factors (as discussed above), but household or infant-specific factors not controlled for by the included variables could continue to bias estimates. Because food recall data is only for the last 24 h, it is possible that the infant occasionally receives some other foods but simply did not the previous day which would result in an underestimate of the effect of exclusive breastfeeding of the infant (18). It has been argued that self-reported recall morbidity data in the DHS, similar to the outcomes represented in this study, systematically under represents morbidity (19), but previous work comparing mother’s self-reported health recall with doctors examination finds that morbidity is reported fairly well (20). We use the DHS morbidity recall data because it is the best available for these purposes, but note this caveat. We also were unable to control for access to sanitary water: a necessary ingredient in infant formula without which our results could be biased upward. Finally, we were unable to include household wealth as a control variable, because it was only included in a subset of the surveys, so we relied on education, work status, and occupation to proxy for household resources. We tested this by conducting analysis on the subset of surveys that contained the wealth variable with and without the wealth variable included. Inclusion of the wealth variable did not substantially alter estimates.

We have quantified relationships between several different types of feeding and health outcomes for a large and geographically diverse set of infants. These findings can be used to inform public health policy related to infant feeding. Mothers without information on breastfeeding guidelines have been reported to be between eight and nine times more likely to introduce supplementary foods too early (15). Providing specific quantified estimates of the burden of these practices can help support decisions to improve feeding practices.

## Author Contributions

Benjamin O. Yarnoff led the research design, statistical analysis, interpretation of results, and manuscript writing. Benjamin T. Allaire assisted in the research design, statistical analysis, interpretation of results, and manuscript writing. Patrick Detzel assisted in the interpretation of results and manuscript writing.

## Conflict of Interest Statement

Research was funded by Nestle Nutrition Institute. The funding organization is an independent institute that is associated with a company that produces infant formula. Patrick Detzel is an employe of Nestlé Research Center.

## Appendix

**Table A1:** **Countries and years in sample**.

Country	Years
Bangladesh	1999
Benin	2006, 2001, 1996
Bolivia	2003, 1998
Cameroon	2004, 1998, 1991
Colombia	2010, 2005, 2000, 1995
Dominican republic	2007, 1999, 1996
Egypt	2008, 2005, 2000, 1996
Ghana	2008, 2003, 1993
India	2005, 1992
Indonesia	2007
Kenya	2008, 2003, 1998, 1993
Madagascar	2008, 2003, 1997, 1992
Mali	2001, 1995
Nepal	2006, 1996
Philippines	2008, 1998, 1993
Senegal	2005
Tanzania	2004, 1999, 1996
Turkey	1998, 1993
Uganda	2006, 2000, 1995
Zambia	2007, 2001, 1996, 1992

**Table A2:** **Feeding type definitions**.

Infant formula	Milk liquids	Non-milk liquids	Solid foods
Commercially produced baby formula	Powdered or tinned milk	Plain water	Baby cereal porridge/gruel Bread, noodles, other foods made from grains
	Fresh milk	Sugar water	Potatoes, cassava, or other tubers
		Juice	Eggs
		Tea or coffee	Meat
			Pumpkin, carrots, squash
			Any dark green leafy vegetables
			Mangoes, papayas, other vitamin A fruits
			Any other fruits
			Liver, heart, other organs
			Fish or shellfish
			Food made from beans, peas, lentils, nuts
			Cheese, yogurt, other milk products
			Oil, fats, butter
			Chocolates, sweets, candies, pastries
			Eggs, fish, poultry
			Any other solid or semi-solid foods
			Food made from wheat, maize, rice, sorghum, or other local grains
